# Soil Microbial Diversity and Community Composition in Rice–Fish Co-Culture and Rice Monoculture Farming System

**DOI:** 10.3390/biology11081242

**Published:** 2022-08-20

**Authors:** Noppol Arunrat, Chakriya Sansupa, Praeploy Kongsurakan, Sukanya Sereenonchai, Ryusuke Hatano

**Affiliations:** 1 Faculty of Environment and Resource Studies, Mahidol University, Nakhon Pathom 73170, Thailand; 2 Department of Biology, Faculty of Science, Chiang Mai University, Chiang Mai 50200, Thailand; 3 Graduate School of Fisheries Science and Environmental Studies, Nagasaki University, 1–14 Bunkyo-machi, Nagasaki 852–8521, Japan; 4 Laboratory of Soil Science, Research Faculty of Agriculture, Hokkaido University, Sapporo 060–8589, Japan

**Keywords:** microbial diversity, microbial community composition, 16s rRNA gene, rice–fish co-culture, rice monoculture

## Abstract

**Simple Summary:**

The integration of fish in rice fields can influence the diversity and structural composition of soil microbial communities. Therefore, soil microorganisms between rice–fish co-culture (RF) and rice monoculture (MC) were compared. The key findings revealed that Actinobacteria, Chloroflexi, Proteobacteria, Acidobacteria, and Planctomycetes were the most dominant taxa across both paddy fields. The most abundant genus in MC belonged to *Anaeromyxobacter*, whereas that in RF was *Bacillus.* Nitrogen fixation, aromatic compound degradation, and hydrocarbon degradation were more abundant in RF. Phosphatase, β-glucosidase, cellulase, and urease enzymes were detected in both paddy fields. However, a 2-year conversion from organic rice to rice–fish co-culture may not be long enough to significantly alter alpha diversity indices.

**Abstract:**

Soil microorganisms play an important role in determining nutrient cycling. The integration of fish into rice fields can influence the diversity and structural composition of soil microbial communities. However, regarding the rice–fish co-culture (RF) farming system in Thailand, the study of the diversity and composition of soil microbes is still limited. Here, we aim to compare the microbial diversity, community composition, and functional structure of the bacterial communities between RF and rice monoculture (MC) farming systems and identify the environmental factors shaping bacterial community composition. Bacterial taxonomy was observed using 16s rRNA gene amplicon sequencing, and the functional structures of the bacterial communities were predicted based on their taxonomy and sequences. The results showed that soil organic carbon, total nitrogen (TN), organic matter, available phosphorous, and clay content were significantly higher in RF than in MC. The most dominant taxa across both paddy rice fields belonged to Actinobacteria, Chloroflexi, Proteobacteria, Acidobacteria, and Planctomycetes. The taxa Nitrosporae, Rokubacteria, GAL15, and Elusimicrobia were significantly different between both rice fields. At the genus level, *Bacillus*, *Anaeromyxobacter*, and HSB OF53-F07 were the predominant genera in both rice fields. The most abundant genus in MC was *Anaeromyxobacter*, whereas RF belonged to *Bacillus*. The community composition in MC was positively correlated with magnesium and sand content, while in RF was positively correlated with pH, TN, and clay content. Nitrogen fixation, aromatic compound degradation, and hydrocarbon degradation were more abundant in RF, while cellulolysis, nitrification, ureolysis, and phototrophy functional groups were more abundant in MC. The enzymes involved in paddy soil ecosystems included phosphatase, β-glucosidase, cellulase, and urease. These results provide novel insights into integrated fish in the paddy field as an efficient agricultural development strategy for enhancing soil microorganisms that increase soil fertility.

## 1. Introduction

Microorganisms play important roles in soil and agricultural ecosystems [1,2]. They are responsible for several processes, such as biomass decomposition, nutrient circulation, and soil formation, which subsequently affect plant growth and production [1,2,3]. In recent years, soil microbial communities have been extensively investigated, as they may reflect soil fertility and ecosystem function [4,5]. Furthermore, the soil microbial community can be used as an indicator to track changes in various land management methods, such as tracking changes in restoration outcomes [6,7] or evaluating agricultural management methods [8].

Integrated rice and fish farming has been practiced in Thailand for more than 200 years by capturing wild seed fish in the rice fields [9]. The rice–fish co-culture (RF) is eulogized for improving ecosystems and alleviating poverty [10] and promoted as increasing biodiversity, reducing fertilizer and pesticide utilization, and contributing to system stability and sustainability [11,12]. Several studies (i.e., [13,14,15]) reported that RF generated the extra production of aquaculture, which increased farmers’ income. Due to fish eating insects, pests, and weeds, the use of pesticides and herbicides can be reduced [16] while organic fertilizers and organic amendments are more applied. These promote the suitable condition for the abundant and diversified population of soil microorganisms, especially bacteria that play a crucial role in soil carbon and nitrogen mineralization. Proteobacteria, Bacteroidetes, Acidobacteria, and Chloroflexi were generally dominant phyla in the paddy soil [17,18,19], which play important roles in soil nutrient cycles [20,21]. Thus, soil microbial communities can be used as an indicator to explain soil health [22].

To date, the scientific knowledge on soil microbial taxonomic and functional composition, and their interactions with environmental factors of integrating fish in paddy fields remain unclear. Therefore, our study was carried out to fill this gap, aiming to (i) compare microbial diversity and community composition between rice monoculture (MC) and RF fields, (ii) identify the environmental factors shaping the bacterial community composition, and (iii) compare the functional structure of the bacterial communities between both study sites. This study can provide scientific knowledge for the development of a rice–fish co-culture farming system.

## 2. Materials and Methods

### 2.1. Description of Study Sites

**Figure 1:**
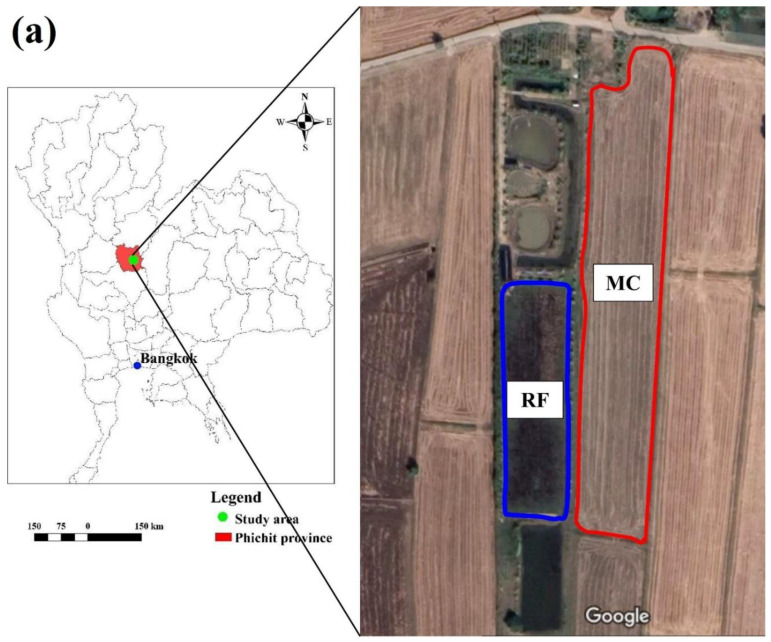

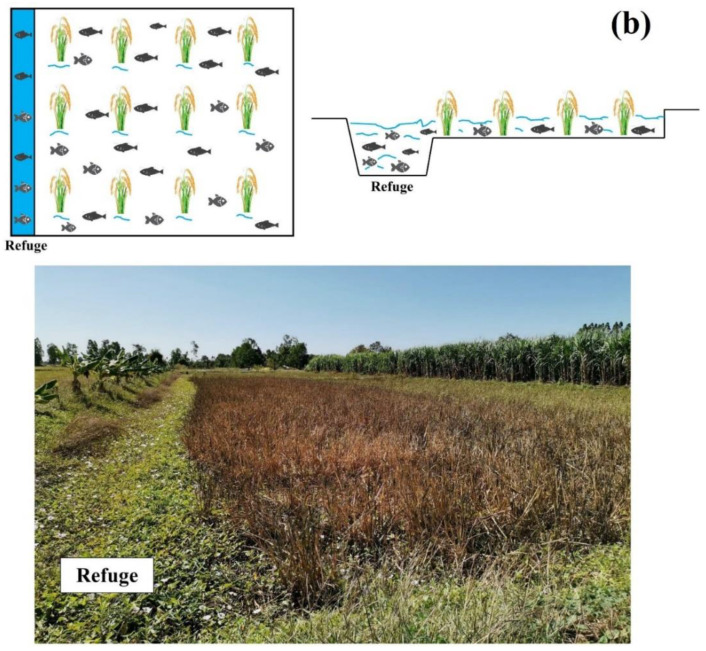
Study areas. (**a**) study sites, (**b**) canal refuge. The aerial image was taken from Google maps on 20 April 2022. The photo was taken on 27 December 2021 by Noppol Arunrat.

The study sites were located in the Samnak Khun Nen Subdistrict, Dong Charoen District, Phichit Province, Lower North of Thailand. The maximum and minimum temperatures were 32.9 and 23.3 °C, respectively, while the average precipitation was 1264.8 mm year^−1^. A rice–fish co-culture farm (16°04′04.1″ N, 100°32′31.1″ E, Figure 1a) which has been producing organic rice for more than 10 years was selected. The International Federation of Organic Agriculture Movements (IFOAM) Standard was first certified in 2016, while the EU/USDA Organic Standard was approved in 2018. The “Riceberry” rice variety was usually grown once per year from August to December. Since 2019, this farm has been raising fish in the paddy field. The main species of fish were common snakehead (*Channa striata*), walking catfish (*Clarias batrachus* (L.)), and Nile tilapia (*Oreochromis niloticus*). The rice bran, vegetable, and fruit residues and cattle manure were applied in the paddy field as the food for the fish and nutrients for rice. The weeds were removed by hand, while biofermented juice was produced from lemongrass, neem leaves, fruits, and vegetables and then mixed with molasses and animal dung (poultry and cattle) to dispose of the insects. The type of rice–fish co-culture field was the canal refuge (Figure 1b). The transplanting method was used for rice planting, which was performed by hand. One-month-old fish were released into the paddy field 30 days after rice planting. The water level in the field was maintained at around 20–30 cm during rice growing. Rice harvesting was performed by hand, and all rice residues were left in the paddy field. Rice yield was approximately 3.6 tons ha^−1^, while the yield of fish was around 300 kg ha^−1^.

For a fair comparison, an adjacent conventional rice farm (16°04′04.6″ N, 100°32′31.9″ E) was selected as the comparison site (Figure 1a). The “RD41” (105 days) or “RD57” (110 days) rice varieties were chosen for planting once a year (August to November). The pregerminated rice seeds were sown by hand (broadcasting method). Then, N, P_2_O_5_, and K_2_O chemical fertilizers were applied, namely 46-0-0 (125 kg ha^−1^) and 16-20-0 (156.3 kg ha^−1^). Glyphosate (48% *w*/*v* SL) and alachlor (48% *w*/*v* EC) were used to kill the weeds, while acephate (75% S) and chlorpyrifos (40% EC) were applied to eliminate the diseases and insects. A harvesting machine was usually used for rice harvesting, then all rice residues were left in the paddy field. The rice yield was approximately 4.7 ton ha^−1^.

### 2.2. Soil Sampling and Measurements

In December 2021, soil samples were collected from the top layer (0–10 cm) of the rice–fish co-culture field and conventional rice field. Five duplicates of soil samples were collected for each field, and 10 soil samples were collected in total. The 50 g soil samples were kept in a cold storage box and brought to the laboratory for the extraction of soil microbial DNA.

Moreover, the soil cores (5.0 cm width × 5.5 cm length) were used to collect the soil samples for soil bulk density measurement and were then measured after 24 h of drying in an oven at 105 °C. The 1 kg soil samples were taken for soil physical and chemical properties analysis.

Soil texture was measured using a hydrometer. Electrical conductivity (ECe) was measured using an EC meter in saturation paste extracts (1:5) [23]. Soil pH was determined using a pH meter in a 1:1 soil-to-water mixture [24]. Available phosphorus (Avail. P) was determined following the molybdate blue method (Bray II extraction) [25]. Available potassium (Avail. K), available calcium (Avail. Ca), and available magnesium (Avail. Mg) were measured using atomic absorption spectrometry (NH4OAc extraction pH 7.0) [26]. Total nitrogen (TN) was measured using the micro-Kjeldahl method. Organic carbon (OC) was analyzed using the Walkley and Black [27] method and converted to organic matter (OM) by multiplying by 1.724. The SOC stock was calculated using the following equation:

*SOCstock* = *OC* × *BD* × *L*, (1)

where *SOCstock* is the soil organic carbon stock (Mg C ha^−1^), *OC* is organic carbon (%), *BD* is soil bulk density (Mg m^−3^), and *L* is soil depth (cm).

### 2.3. DNA Extraction and Amplicon Sequencing of 16s rRNA Gene

DNA was extracted from 0.25 g of soil using DNeasy PowerSoil Pro DNA Kit (Qiagen, Germantown, MD, USA) following the manufacturer’s instructions. The extracted DNA was subjected to amplicon library preparation and sequencing. Briefly, PCR amplification, targeting the V3–V4 variable of the 16s rRNA gene, was performed using the universal primers 341F (5′-CCTAYGGGDBGCWSCAG) and 805R (5′-GGA CTACNVGGGTHTCTAAT) (Klindworth et al., 2013). The amplicons were then sequenced on the Illumina Miseq platform (2 × 250 bp). The amplification and sequencing steps were run by Omics Sciences and Bioinformatics Center (Chulalongkorn University, Bangkok, Thailand).

### 2.4. Sequencing Analysis and Microbial Taxonomic Identification

The raw sequence dataset was analyzed with QIIME 2 v. 2021.8 [28]. The 16s rRNA primers were trimmed from forward and reverse reads using cutadapt [29]. The trimmed sequences were quality-filtered (MaxEE = 2; no ambiguous nucleotide) and merged (minimum overlap = 12 nucleotides), and chimeras were removed using the DADA2 plugin [30]. The high-quality sequence was clustered at 97% sequence identity into operational taxonomic units (OTUs) using the VSEARCH plugin [31,32]. Representative sequences of each OTU were taxonomically identified against the Silva v.138 database [33,34]. To eliminate potential sequencing error, rare OTUs (singletons, doubletons, and tripletons) were removed from the dataset. After that, the number of reads that remained in each sample was randomly subsampled and normalized to the smallest number of reads per sample (24,676 reads/sample), to avoid sequencing depth bias, using rarefy was implemented in QIIME 2. These normalized datasets were used for further analysis.

### 2.5. Functional Prediction

Microbial associated functions were predicted using FAPROTAX [35,36] and PICRUSt2 [37]. The FAPROTAX predicted the ecologically relevant function of each taxon based on data of the cultured taxa. For example, if all cultured taxa of a bacterial genus were identified as nitrogen-fixing bacteria, all uncultured members of that genus will also be identified as nitrogen-fixing bacteria. On the other hand, PICRUSt2 predicted potential functions based on gene sequences presented in each taxon. In this study, the PICRUSt function was emphasized as enzyme activities that were potentially performed by the detected taxon. These functional analyses were performed following the instructions on the FAPROTAX (http://www.loucalab.com/archive/FAPROTAX/lib/php/index.php?section=Home accessed on 27 March 2022) and PICRUSt (https://github.com/picrust/picrust2/wiki accessed on 27 March 2022) webpages.

### 2.6. Statistical Analysis

Statistical analysis was performed on PAST [38] and R statistical software [39]. Independent *t*-tests were employed for comparison of soil physicochemical properties between monoculture rice fields and rice–fish co-culture fields. The correlations among the physicochemical variables were observed via Pearson’s correlation matrixes. Differences in the relative abundance of microbial taxa detected in the two study sites were indicated using the linear discriminant analysis (LDA) effect size (LEfSe) [40]. Taxa with significant *p*-values (*p* < 0.05) and LDA score ≥ 2 were considered differentially abundant taxa. The LEfSe analysis was performed on an online interface of the Huttenhower lab Galaxy server (http://huttenhower.sph.harvard.edu/galaxy accessed on 27 March 2022). Alpha diversity, including observed OTU richness, Shannon, and Simpson indices were estimated using the diversity indices function in PAST. Differences in the alpha diversity indices between the two study sites were tested via t-test. Beta diversity, representing community composition, was analyzed using non-metric multidimensional scaling (NMDS) ordination based on Bray–Curtis dissimilarity, which was computed using the metaMDS function in the vegan R package. Permutational MANOVA (PERMANOVA) was used to the calculated difference between the two community compositions using the adonis function. The influence of soil properties on soil bacterial community composition was estimated by correlation analysis. The correlations were calculated using the envfit function in the vegan R package, and the *p*-values were corrected by Bonferroni’s correction using the p.adjust function in the stat R package. The NMDS ordination with significantly correlated soil parameters was plotted using the ggplot2 R package. Bacterial-associated functions, predicted by both FAPROTAX and PICRUSt2, were visualized as extended bar plots in STAMP software. Statistical differences between each function were tested via *t*-test, and all *p*-values were corrected using Bonferroni’s correction. Functional compositions were analyzed following the community composition (beta diversity) analysis as described above.

## 3. Results

### 3.1. Soil Physicochemical Properties in Rice Monoculture and Rice–Fish Co-Culture Fields

The soil samples, both from the rice monoculture (MC) and the rice–fish co-culture fields (RF), were silty clay. However, significant variances in soil physiochemical properties were found (Table 1). Lower acidity (6.0 ± 0.2, *p* < 0.01), bulk density (1.4 ± 0.02 Mg m^−3^, *p* < 0.05), ECe (0.4 ± 0.01 dS m^−1^, *p* < 0.01), available Ca (2279.0 ± 90.0 mg kg^−1^, *p* < 0.01), available Mg (175.1 ± 3.6 mg kg^−1^, *p* < 0.01), and %Sand (10.1 ± 0.8, *p* < 0.01) were easily observed in the RF field. Meanwhile, the RF soils also contained significantly higher contents of OM fraction (3.4% ± 0.2, *p* < 0.01), SOC (80.9 ± 3.5 Mg C ha^−1^, *p* < 0.01), TN (0.5% ± 0.02, *p* < 0.01), available P (20.0 ± 0.9 mg kg^−1^, *p* < 0.01), and %Clay content (46.3 ± 0.9 mg kg^−1^, *p* < 0.01). However, there was no significant difference in available K content or %Silt content between the two sampling sites. Negative correlations between SOC stock and %Silt content were found in MC (r = -0.887, *p* < 0.05). In RF, negative correlations were found between SOC and ECe (r = –0.904, *p* < 0.05) as well as between available K and %Silt content (r = –0.992, *p* < 0.05). Moreover, total nitrogen positively correlated with %Sand content (r = 0.917, *p* < 0.05) (Table 2).

### 3.2. General Overview of the Sequencing Analysis

**Figure 2:**
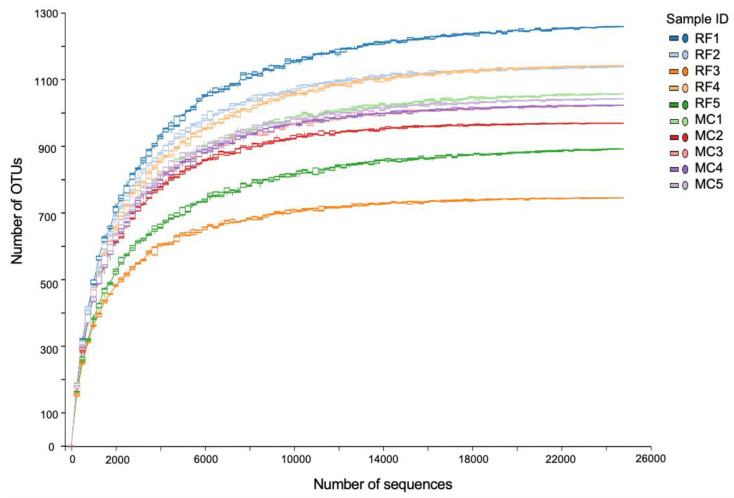
Rarefaction curve of observed microbial OTUs detected in monoculture (MC) and rice–fish (RC) fields.

A total of 337,778 high quality and abundance readings, representing 4597 OTUs, were derived from this study. After normalization, 4582 OTUs remained. Rarefaction curves of the detected OTUs derived from both MC and RF samples were flattened at the analysis sequencing depth (24,676 reads/sample), indicating that the detected OTUs were sufficient to represent the microbial community in each sample (Figure 2).

### 3.3. Taxonomic Distribution and Differential Abundance of Soil Bacteria in Rice Monoculture and Rice–Fish Co-Culture Fields

**Figure 3:**
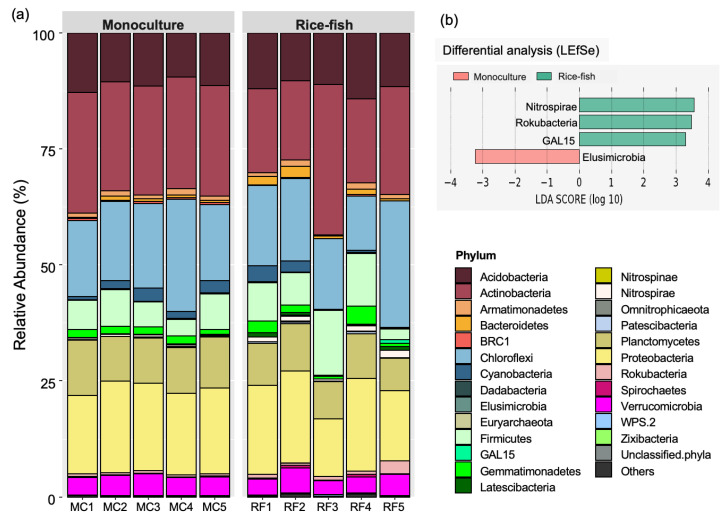
Taxonomic distribution at phylum level. (**a**) The relative abundance of microbial phyla in each sample. (**b**) LEfSe analysis of differential taxonomy abundance at phylum level between the two study sites. The orange and blue horizontal bars represent the taxa enriched in monoculture and rice–fish fields, respectively.

According to microbial analysis based on the 16s rRNA gene, microbial taxa detected in this study were classified into 47 phyla, 128 classes, 235 orders, 318 families, 479 genera and 4582 OTUs. Taxonomic distribution of the abundant bacteria (total relative abundance > 0.1%) is presented in Figure 2. The most dominant taxa across all samples belonged to Actinobacteria (MC = 22.78%, RF= 24.17%, on average), followed by Chloroflexi (MC = 18.44%, RF= 17.77%), Proteobacteria (MC = 18.25%, RF= 17.28%), Acidobacteria (MC = 11.16%, RF= 11.88%), and Planctomycetes (MC = 10.44%, RF= 8.76%) (Figure 3a). Taxa that were significantly different between the two sites were Nitrosporae, Rokubacteria, GAL15, and Elusimicrobia. The latter was enriched in RF, whereas the other three were enriched in MC (Figure 3b).

**Figure 4:**
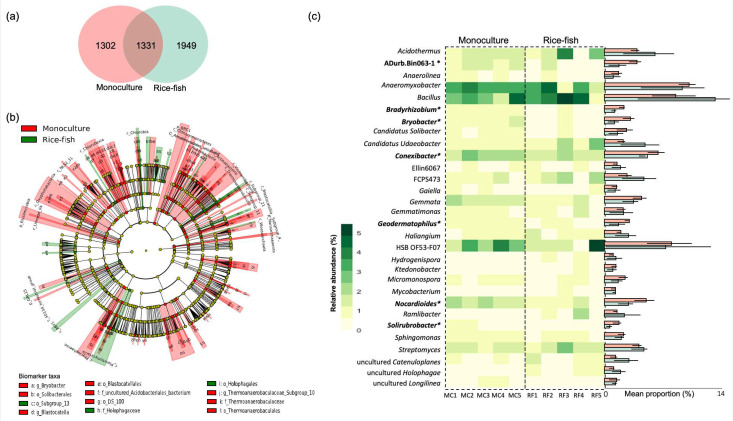
Taxonomic differences of bacteria in monoculture and rice–fish co-culture fields. (**a**) The Venn diagram shows the number of OTUs found in monoculture (red) and rice–fish (green) fields. (**b**) The cladogram shows differential abundance taxa among the two rice fields. More information on the differential taxa is provided in Supplementary Figures S1 and S2. (**c**) Heatmap shows the relative abundance of the thirty most abundant microbial genera detected in each sample. The bar plot presents the mean relative abundance of the microbial genera detected in monoculture (orange) and rice–fish fields (green). Genus names with an asterisk are statistically different between the two sites (*p* < 0.05). MC: Monoculture, RF: rice–fish.

The difference in the microbial community was more noticeable at a deeper taxonomic level, as shown in Figure 4. More than 50% of all detected OTUs were found uniquely in one of the two study sites (Figure 4a). According to the LEfSe analysis at the phylum to genus level, a total of 135 differentially abundant taxa (*p* < 0.05; LDA score > 2) were detected, 111 of which were more abundant in MC than RF and 24 of which were more abundant in RF than MC (Figure 4b, Figure S1). At the genus level, *Bacillus*, *Anaeromyxobacter*, and HSB OF53-F07 were the predominant genera in both rice fields. The most abundant genus in MC was *Anaeromyxobacter*, whereas that in RF was *Bacillus* (Figure 4c). Seven out of the top 30 most prevalent genera, for example, *Bradyrhizobium*, *Bryobacter*, *Conexibacter*, *Nocadiodides,* and *Solirubrobacter,* were significantly more abundant in MC than in RF (Figure 4c). However, some low-abundance genera, such as *Chlorobaculum*, *Niastella,* and *Vicinamibacter*, were more abundant in RF than in MC (Figure S1).

### 3.4. Richness, Diversity, Community Composition, and Their Correlation to Soil Properties

**Figure 5:**
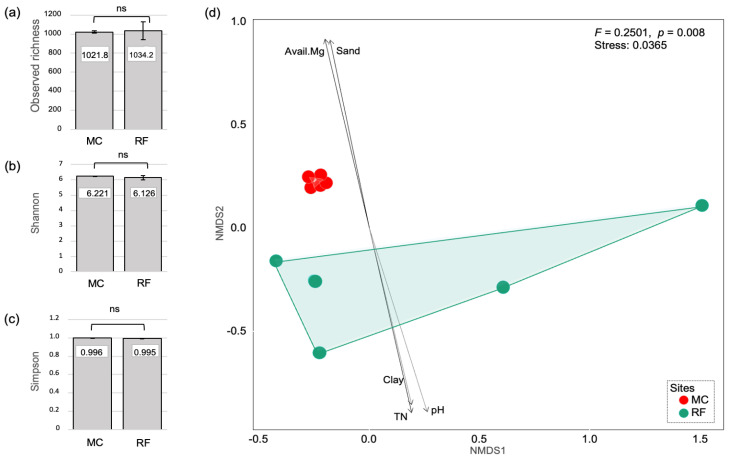
Bacterial diversity and community composition. Bar plot shows (**a**) OTU richness, **(b)** Shannon’s, and **(c)** Simpson’s indices measured in monoculture and rice–fish fields. **(d)** NMDS ordination, based on the Bray–Curtis dissimilarity measure, presents the community composition of soil microbes in the two study sites. MC: Monoculture, RF: rice–fish.

Alpha diversity, reflecting richness and diversity, was indicated by observed OTU richness, Shannon, and Simpson indices. As shown in Figure 5, all alpha diversity indices were slightly higher in MC than in RF, but no significant difference was found between the two sites (*p* > 0.05, Figure 5a–c). On the contrary, beta diversity, presented by NMDS ordination with Bray–Curtis distance, showed a separated community between MC and RF (Figure 5(d)). This indicated that the community composition of bacteria in MC was different from that in RF. The difference was confirmed by PERMANOVA test (*F* = 0.251, *p* = 0.008).

The correlations between soil properties and bacterial community composition are shown in Table 3; 5 out of 12 measured parameters were significantly correlated with bacterial community composition in both study sites. Whilst the community composition in MC was positively correlated with Mg and sand, the same in RF was positively correlated with pH, TN, and clay (Figure 5). Mg was the most correlated factor (r^2^ = 0.880), following closely by Sand (r^2^ = 0.866), pH (r^2^ = 0.857) and TN (r^2^ = 0.834) (Table 3).

### 3.5. Predictive Function

**Figure 6:**
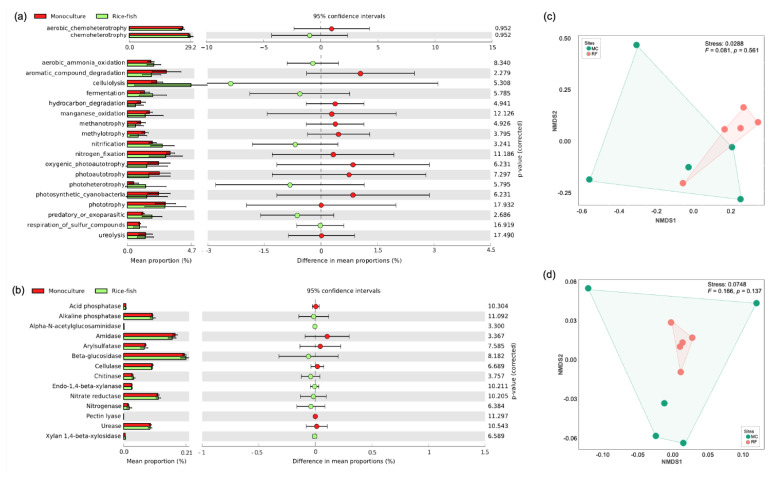
Predictive functions. Extended bar plots show mean and difference in mean proportions of microbial function predicted by (**a**) FAPROTAX and (**b**) PICRUST2. Functions overrepresented in the monoculture (red) have a positive difference in the proportion, whereas functions overrepresented in the rice–fish (green) have a negative difference. NMDS ordinations based on the Bray–Curtis dissimilarity measure show the composition of bacterial functions, predicted by (**c**) FAPROTAX and (**d**) PICRUST2, in monoculture (red) and rice–fish field (green). MC: Monoculture, RF: rice–fish.

Totals of 807 (17.61%) and 4532 (98.90%) OTUs were assigned to at least one function of the 63 ecologically relevant functions and 2238 enzymes, respectively. For FAPROTAX analysis, Cellulolysis, nitrification, ureolysis, phototrophy, nitrogen fixation, aromatic compound degradation, and hydrocarbon degradation, were found among the top 20 most abundant functions (Figure 6a). While the first four functional groups were more abundant in MC, the last three groups were more abundant in RF. However, no significant change was detected in any of those functions (*p* > 0.05), which is consistent with the results from the PICRUSt analysis. Enzymes involved in soil systems, such as phosphatase, β-glucosidase, cellulase, and urease, were presented in this study. As shown in Figure 6b, no significant difference was found between the enzymes detected in MC and RF (*p* > 0.05). The functional potential structures, created from all detected ecologically relevant functions and enzymes, were shown in Figure 6c and Figure 6d, respectively. Based on NMDS ordination and PERMANOVA analysis, no significant difference was detected (*p* > 0.05) between the functional structures in MC and RF.

## 4. Discussion

### 4.1. RF Farming System Can Increase Soil Nutrients

Our results (Table 1) reveal that the rice–fish co-culture system significantly increased SOC stock and TN similarly to other studies [41,42,43,44], which found that the rice–fish co-culture system can potentially increase the content of organic carbon and nitrogen in the soil through its mineralization of organic matter. This is due to the uneaten and excess feed as well as excreta produced during fish growth increasing SOC content and TN, which is consistent with the findings in the rice–crayfish–turtle co-culture by Li et al. [45] and rice–crayfish farming system by Si et al. [46]. They also revealed that crayfish and turtle feeding, molting, and excretion could increase the SOC and TN, while these aquatic animals help increase soil permeability by penetrating the soil surface in the paddy field. The supplies of some available elements (i.e., Ca and Mg) in the RF farming system were significantly lower than in MC (Table 1), which were possibly shaped by its divalent charge fraction on the ECe [47], following the lower ECe value in RF compared to MC (Table 1). The rice–fish co-culture system, however, shows a higher content of available P (Table 1), while in rice monoculture, it generally decreased due to the long-term cultivation. Slowly cycling P and labile P in soil increasing with time was also previously reported in the rice-frog-fish culture soil [48]. Higher clay content and organic matter were found in RF compared with MC due to a higher amount of organic amendments being added. Hassink [49] reported that clay particles could absorb organic matter and protect it from microbial decomposition.

### 4.2. Microbial Diversity and Community Composition under Rice–Fish Co-Culture and Rice Monoculture

To our knowledge, this study was among a few studies investigating the differentiation of both community and functional potential structures of soil bacteria in rice monoculture fields, compared with rice–fish co-culture fields. Here, we found that although the alpha diversity of bacteria did not differ significantly between MC and RF, the community composition did. Even though the alpha diversity indices, which present observed richness, Shannon’s, and Simpson’s indices, differed between the two sites, the differences were minor and did not reveal a significant difference. This result is consistent with previous studies on other rice co-culture fields, including rice–frog (Yi et al., 2019), rice–crab [19], and rice–fish fields [7,45]. Whilst Zhao et al. [7] reported that microbial diversity in rice–fish co-culture fields was similar to those in monoculture rice fields in the first year of cultivation and substantially changed after 5 years, Li et al. [45] found that it took up to 12 years to see the differences in rice–fish–turtle fields. Here, we showed that changes were still not found after three years of rice–fish cultivation. However, when it comes to community composition, all studies [7,45,50], including this one, found significant differences between co-culture and monoculture rice fields. A total of 135 differentially abundant taxa were found in both the MC and RF sites, four of which were among the abundant phyla and seven of which were among the top 30 most abundant genera, respectively. These results implied that three years of rice–fish co-culture agriculture, which was implemented after long-term monoculture rice cultivation, had a significant impact on microbial community composition, while diversity remained unchanged.

As shown in Figure 3a, the most dominant taxa in paddy fields (both RF and MC farming systems) belonged to Actinobacteria, Chloroflexi, Proteobacteria, Acidobacteria, and Planctomycetes. Meanwhile, all alpha diversity indices showed no significant difference between the two sites. This is consistent with the study of Jiang et al. [19], who found that the most abundant phyla in paddy soil and ditch sediment under the rice–crab co-culture system were Proteobacteria, Bacteroidetes, and Chloroflexi, while alpha diversity of bacterial diversity in paddy soil and ditch sediment was similar. The phylum Actinobacteria, which contributes to the turnover of the complex biopolymers and plant residue decomposition [51,52] by producing various carbon cycling enzymes [53], was the most dominant taxa in both RF and MC sites. The phylum Chloroflexi, which involves nitrification was the second-most dominant that was detected in RF and MC sites [54]. The phylum Proteobacteria is usually classified as “copiotrophs” (R-strategists), which indicates that its members are more abundant and have high growth rates under nutrient-rich conditions [55]. It plays a key role in OM decomposition, produces many types of glycosyl hydrolases, and is involved in nitrogen fixation, which promotes plant growth [1]. The phylum Acidobacteria is involved in the carbon cycle of humus decomposition [56] and adjusts soil pH [57]. Members of the bacterial phylum Planctomycetes can act as slow-acting degraders of various biopolymers, cellulose, and chitin. They can degrade exopolysaccharides produced by other bacteria [58]. García-Orenes et al. [59] reported that some members of the phylum Planctomycete, such as *Blastopirellula,* are good indicators of soil fertility because they respond to the applications of manure or fertilizers faster than they react to soil physicochemical properties.

Nitrosporae, Rokubacteria, GAL15, and Elusimicrobia taxa were significantly different between the RF and MC fields (Figure 3b). This result indicated that the structure of the soil bacterial community was significantly changed after integrating fish into the paddy field. The phylum Nitrospirae plays a crucial role of decomposing soil mineral nitrogen and improving nutrient availability [60], and its functions can enhance crop productivity [61]. At the genus level, *Bacillus*, *Anaeromyxobacter*, and HSB OF53-F07 were found in both rice fields. The most abundant genus in MC was *Anaeromyxobacter*, whereas *Bacillus* was the most dominant in RF (Figure 4c). *Bacillus* plays multiple functions in the soil ecosystem for nutrient cycling, which is involved in nitrogen fixation, phosphorus nutrition, and potassium solubilization that promotes plant growth [62]. Similarly, *Anaeromyxobacter* can fix and assimilate N_2_ gas via its nitrogenase [63]. This is why soil nutrients (TN, Avail. P, and Avail. K) were higher in RF than in MC soil (Table 1).

Soil physicochemical properties have a great influence on bacterial community composition [7,50,64,65]. Our findings indicated that the different soil management practices of RF and MC farming systems cause a significant difference in soil physicochemical properties, resulting in the different composition of soil bacterial communities and their metabolic functions (Figure 5 and Table 3). This is in line with the study of Viruel et al. [66], who reported that changes in SOC stock, TN, and pH cause the changes in soil bacterial communities and metabolic functions in farming systems of the semi-arid Chaco region, Argentina. Hartmann et al. [67] found that soil pH, SOC, and TN were the main predictors of bacterial community structure in long-term organic and conventional farming. As presented in Figure 5 and Table 3, we found that Mg, TN, pH, and soil texture (percent of sand and clay) were the main factors determining the community composition of bacteria in MC and RF. TN and pH have previously been identified as the key factors that influence the microbial community in rice co-culture fields [7,50]. While Mg was rarely measured in earlier works, this study found that it was one of the most important elements affecting community composition in rice fields. Hou et al. [68] found a strong effect of Mg on bacterial nitrification, which is in line with the study of Zhang et al. [69], who reported that the appropriate concentrations of Mg^2+^ could promote nitrification activity in the soil.

In addition, this study also showed the functional potential structure of the bacterial community, based on FAPROTAX and PICRUSt2, in MC and RF (Figure 6). Interestingly, despite differences in community composition between MC and RF, no difference in functional potential structure was found. Furthermore, there was no difference in the abundance of the individual function predicted by either tool. Recently, Chen et al. [70] demonstrated on global soil metagenomic data that microbial functional structure could remain stable while taxonomic diversity and composition changed, which is consistent with our findings. We reveal that, whilst bacterial community composition changed during the transition from monoculture to rice–fish co-culture, the functional structure could be maintained, and this was the first study to report such results on monoculture and rice–fish fields. However, it should be noted that the limitation of the prediction tools was that only taxa or genes from the databases were functionally assigned [35,36,37,71]. Regardless, using both tools to estimate the availability and abundance of genes or functions within the community could provide more insight into information on the complex community in soil.

## 5. Conclusions

This study shows that soil physicochemical properties (SOC, TN, OM, Avail. P, and clay content) were significantly higher in RF than in MC sites. The most dominant taxa across both paddy rice-farming systems belonged to Actinobacteria, Chloroflexi, Proteobacteria, Acidobacteria, and Planctomycetes. The taxa Nitrosporae, Rokubacteria, GAL15, and Elusimicrobia were significantly different between the RF and MC sites. At the genus level, *Bacillus*, *Anaeromyxobacter*, and HSB OF53-F07 were the predominant genera in both rice fields. The most abundant genus in MC was *Anaeromyxobacter*, that in RF was *Bacillus.* All alpha diversity indices between the RF and MC sites were not significantly different. The community composition in MC was positively correlated with Mg and sand, while in RF was positively correlated with pH, TN, and clay. Nitrogen fixation, aromatic compound degradation, and hydrocarbon degradation were more abundant in RF, while cellulolysis, nitrification, ureolysis, and phototrophy functional groups were more abundant in MC. The enzymes involved in paddy soil ecosystems included phosphatase, β-glucosidase, cellulase, and urease, but no significant difference was detected between MC and RF fields. However, more observation fields and long-term monitoring are necessary to conclusively confirm our findings in this study.

## Figures and Tables

**Table 1:** Comparison of soil physicochemical properties of rice monoculture and rice–fish co-culture fields.

Soil Properties	Rice Monoculture	Rice–fish Co-Culture	T	Sig.
pH (1:2.5)	4.7 ± 0.3	6.0 ± 0.2	8.031	**
BD (Mg m^−3^)	1.4 ± 0.02	1.4 ± 0.02	−2.414	*
OM (%)	2.1 ± 0.4	3.4 ± 0.2	6.997	**
SOC (Mg C ha^−1^)	51.0 ± 9.2	80.9 ± 3.5	13.878	**
TN (%)	0.3 ± 0.01	0.5 ± 0.2	15.637	**
ECe (dS m^−^^1^)	1.0 ± 0.4	0.4 ± 0.01	−3.636	**
Avail. P (mg kg^−1^)	13.6 ± 1.9	20.0 ± 0.9	6.928	**
Avail. K (mg kg^−1^)	162.8 ± 6.2	170.0 ± 4.1	2.182	NS
Avail. Ca (mg kg^−1^)	2554.4 ± 85.2	2279.0 ± 90.0	−4.967	**
Avail. Mg (mg kg^−1^)	225.0 ± 5.6	175.1 ± 3.6	−16.709	**
Sand (%)	17.4 ± 0.9	10.1 ± 0.8	−14.141	**
Silt (%)	42.0 ± 1.3	43.6 ± 1.0	2.123	NS
Clay (%)	40.6 ± 0.9	46.3 ± 0.9	10.486	**
Soil texture	Silty Clay	Silty Clay	-	-

*, ** indicate statistically significant with *p*-value < 0.05 and < 0.01, respectively. NS: No significant, BD = bulk density, OM = organic matter, TN = total nitrogen, ECe = electrical conductivity, CEC = cation exchange capacity, Avail. P = available P, Avail. K = available K, Avail. Ca = available Ca, Avail. Mg = available Mg.

**Table 2:** Pearson’s correlation matrixes of soil properties in rice–fish co-culture (n = 5, white area) and rice monoculture fields (n = 5, grey area).

Soil Properties	pH	BD	SOC	TN	EC_e_	P	K	Ca	Mg	%Sand	%Silt	%Clay
**pH**		−0.718	0.391	−0.387	−0.086	−0.302	−0.613	0.275	−0.401	0.061	−0.056	0.010
**BD**	−0.562		0.106	−0.015	−0.160	0.558	0.216	−0.662	0.204	−0.197	−0.313	0.537
**SOC**	−0.316	−0.187		−0.787	−0.485	0.744	−0.367	−0.293	0.328	0.247	**−0.887**	0.796
**TN**	−0.373	0.775	−0.454		−0.157	−0.434	0.773	−0.226	−0.516	−0.709	0.871	−0.361
**ECe**	−0.087	0.524	**−0.904**	0.741		−0.565	−0.493	0.803	0.231	0.626	0.180	−0.770
**P**	−0.101	−0.515	−0.243	−0.316	0.149		0.194	−0.549	0.587	0.121	−0.819	0.831
**K**	0.258	0.618	−0.266	0.512	0.307	−0.868		−0.452	−0.026	−0.543	0.377	0.056
**Ca**	0.700	−0.745	0.046	−0.277	−0.323	0.049	−0.120		0.292	0.733	0.128	−0.808
**Mg**	0.474	0.198	−0.551	0.618	0.498	−0.435	0.668	0.445		0.791	−0.681	0.069
**%Sand**	−0.126	0.811	−0.618	**0.917**	0.808	−0.446	0.744	−0.333	0.691		−0.546	−0.274
**%Silt**	−0.333	−0.559	0.341	−0.535	−0.355	0.827	**−0.992**	0.022	−0.756	−0.762		−0.656
**%Clay**	0.631	0.051	0.090	−0.090	−0.258	−0.818	0.774	0.295	0.468	0.173	−0.770	

All values in bold print are significant (*p* < 0.05). BD = bulk density, OM = organic matter, TN = total nitrogen, ECe = electrical conductivity, CEC = cation exchange capacity, P = available P, K = available K, Ca = available Ca, Mg = available Mg.

**Table 3:** Pearson’s correlation between microbial community composition and soil properties.

Parameter	NMDS1	NMDS2	*r*	*p*-Value
pH	0.284	−0.959	0.857	0.024 *
BD	−0.523	0.853	0.697	0.108
OM	0.286	−0.958	0.762	0.084
TN	0.211	−0.978	0.834	0.012 *
ECe	−0.238	0.971	0.563	0.516
Avail. P	0.266	−0.964	0.662	0.240
Avail. K	0.176	−0.984	0.261	1.000
Avail. Ca	−0.088	0.996	0.649	0.240
Avail. Mg	−0.214	0.977	0.880	0.024 *
Sand	−0.190	0.982	0.866	0.036 *
Silt	0.104	−0.995	0.460	1.000
Clay	0.220	−0.976	0.764	0.048 *

* Indicate significant parameters (*p* < 0.05). BD = bulk density, OM = organic matter, TN = total nitrogen, ECe = electrical conductivity, CEC = cation exchange capacity, Avail. P = available P, Avail. K = available K, Avail. Ca = available Ca, Avail. Mg = available M.

## Data Availability

Raw sequence data generated for this study are available in the Sequence Read Archives (SRA) of the National Center for Biotechnology Information (NCBI) under BioProject accession number: PRJNA819169.

## Supplementary Materials

The following supporting information can be downloaded at: https://www.mdpi.com/article/10.3390/biology11081242/s1, Figure S1: LEfSE analysis from phylum to genus level. The cladogram shows differential abundance in taxa (*p* > 0.05, LDA score ≥ 2) between monoculture rice fields and rice–fish co-culture fields. Taxa in red are significantly enriched in the monoculture, whereas taxa in green are significantly enriched in the rice–fish field. Figure S2: Bar plot shows differential abundance taxa with effect size (LDA score). Taxa in red are significantly enriched in the monoculture, whereas taxa in green are significantly enriched in the rice–fish field.
