# Supplementary material for: Soil Microbial Diversity and Community Composition in Rice–Fish Co-Culture and Rice Monoculture Farming System
Source: Biology (Basel). 2022 Aug 20;11(8):1242. doi: 10.3390/biology11081242 (PMC9404718; doi:10.3390/biology11081242)
Supplement: Supplementary file 1 [file biology-11-01242-s001.zip › biology-1874237-supplementary.pdf]

Supplementary Materials

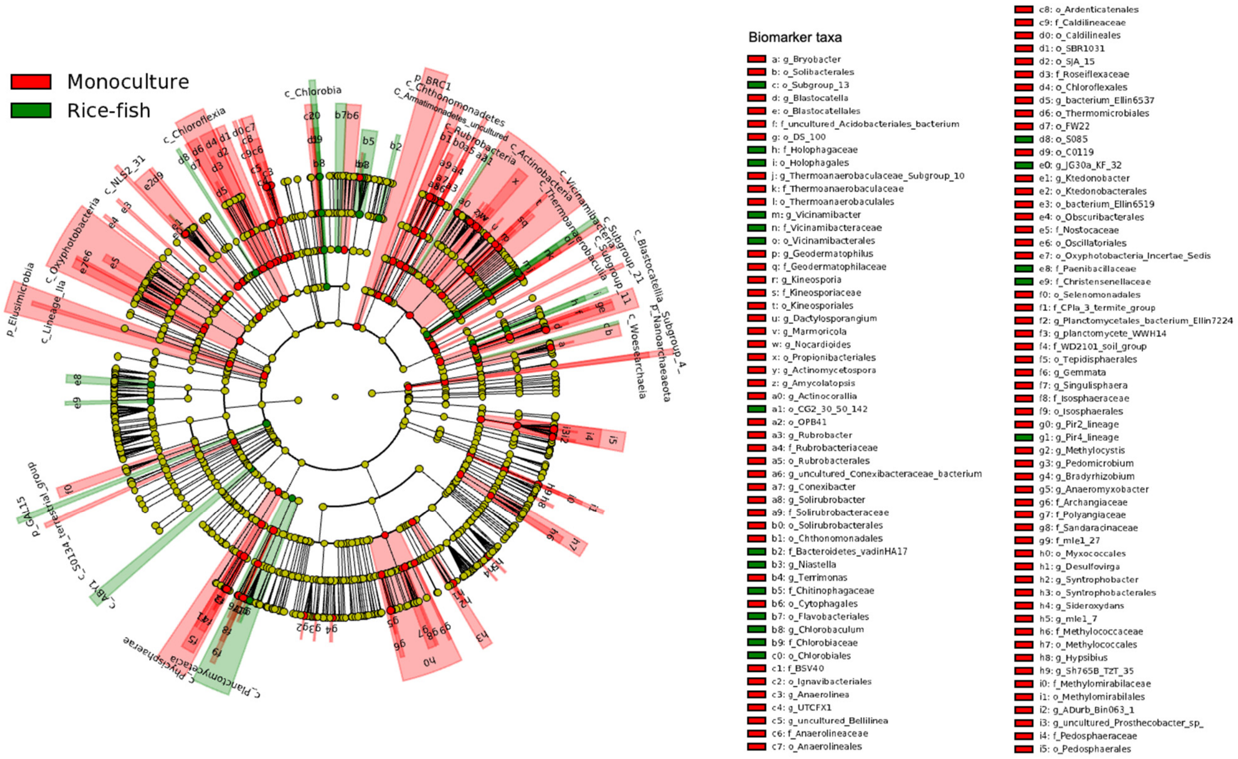

Figure S1 LEfSE analysis from phylum to genus level. Cladogram shows differential abundance taxa ($p > 0.05$, LDA score ≥ 2) among monoculture rice fields and rice-fish co-culture fields. Taxa in red are significantly enriched in the monoculture, whereas taxa in green are significant enriched in the rice-fish field.

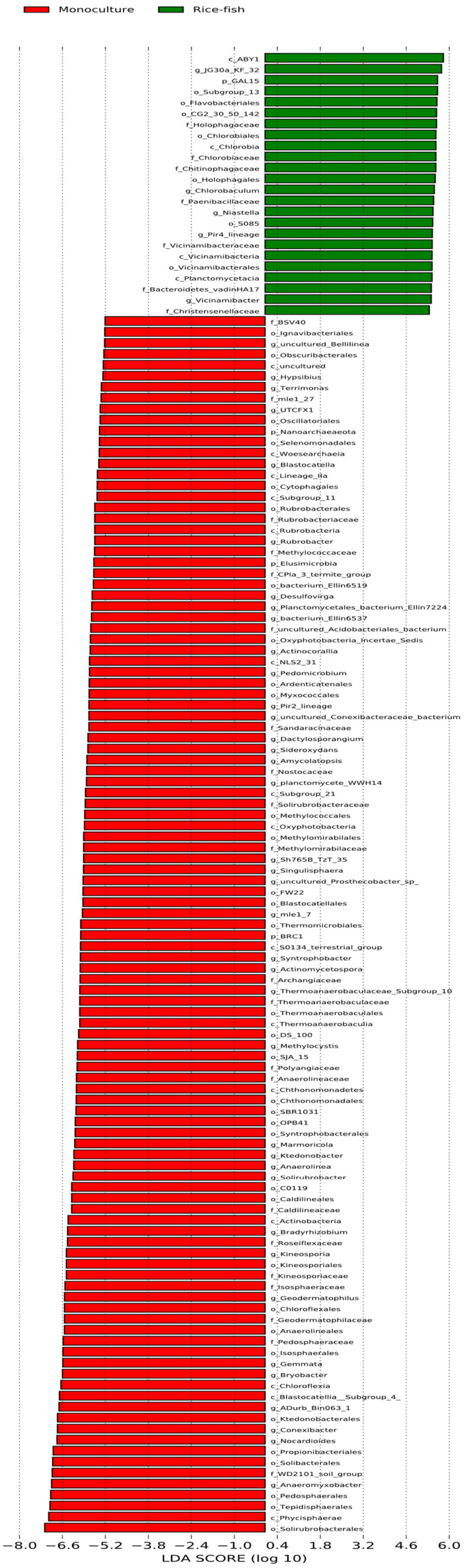

Figure S2 Bar plot shows differential abundance taxa with effect size (LDA score). Taxa in red are significantly enriched in the monoculture, whereas taxa in green are significantly enriched in the rice-fish field.
